# RhoA/Rho‐kinases in asthma: from pathogenesis to therapeutic targets

**DOI:** 10.1002/cti2.1134

**Published:** 2020-04-29

**Authors:** Yan Zhang, Arjun Saradna, Rhea Ratan, Xia Ke, Wei Tu, Danh C Do, Chengping Hu, Peisong Gao

**Affiliations:** ^1^ Division of Allergy and Clinical Immunology Johns Hopkins University School of Medicine Baltimore MD USA; ^2^ Department of Respiratory Medicine Xiangya Hospital Central South University Changsha China; ^3^ Division of Pulmonary Critical Care and Sleep Medicine State University of New York at Buffalo Buffalo NY USA; ^4^ Department of Otorhinolaryngology First Affiliated Hospital of Chongqing Medical University Chongqing China; ^5^ Department of Respirology and Allergy Third Affiliated Hospital of Shenzhen University Shenzhen China

**Keywords:** airway inflammation, airway remodelling, asthma, RhoA, Rho‐kinase, therapy

## Abstract

Asthma is a chronic and heterogeneous disease characterised by airway inflammation and intermittent airway narrowing. The key obstacle in the prevention and treatment of asthma has been our incomplete understanding of its aetiology and biological mechanisms. The ras homolog family member A (RhoA) of the Rho family GTPases has been considered to be one of the most promising and novel therapeutic targets for asthma. It is well known that RhoA/Rho‐kinases play an important role in the pathophysiology of asthma, including airway smooth muscle contraction, airway hyper‐responsiveness, β‐adrenergic desensitisation and airway remodelling. However, recent advances have suggested novel roles for RhoA in regulating allergic airway inflammation. Specifically, RhoA has been shown to regulate allergic airway inflammation through controlling Th2 or Th17 cell differentiation and to regulate airway remodelling through regulating mesenchymal stem cell (MSC) differentiation. In this review, we evaluate the literature regarding the recent advances in the activation of RhoA/Rho‐kinase, cytokine and epigenetic regulation of RhoA/Rho‐kinase, and the role of RhoA/Rho‐kinase in regulating major features of asthma, such as airway hyper‐responsiveness, remodelling and inflammation. We also discuss the importance of the newly identified role of RhoA/Rho‐kinase signalling in MSC differentiation and bronchial epithelial barrier dysfunction. These findings indicate the functional significance of the RhoA/Rho‐kinase pathway in the pathophysiology of asthma and suggest that RhoA/Rho‐kinase signalling may be a promising therapeutic target for the treatment of asthma.

## Graphical abstract

We evaluate recent advances in cytokine and epigenetic regulation of RhoA/Rho‐kinase and the role of RhoA/Rho‐kinase in regulating major clinical features of asthma, such as airway hyper‐responsiveness, remodelling and inflammation. The newly identified roles of RhoA/Rho‐kinase signalling in mesenchymal stem cell differentiation and bronchial epithelial barrier dysfunction are also discussed. We suggest that RhoA/Rho‐kinase signalling may be a promising therapeutic target for the treatment of asthma.

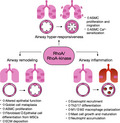

## Introduction

Asthma is a serious chronic and heterogeneous inflammatory disease, with an increasing prevalence and approximately 300 million asthmatics worldwide.^1^ Although the reason for the increased prevalence is unclear, environmental allergens (e.g. house dust mite, cockroach and mouse) and pollutants (e.g. particulate matter [PM]) as a result of increasing urbanisation may be major risk factors contributing to the development of asthma.^2^ Asthma is characterised by reversible airway obstruction, airway hyper‐responsiveness, mucus production, and inflammation and remodelling of the airways.^3^, ^4^ Clinically, asthma mainly presents as coughing, wheezing and shortness of breath. There are at least two distinct groups of asthma patients, one with higher levels of T helper 2 (Th2) cytokines and one with lower levels (non‐Th2 type).^5^ Th2 cytokines, interleukin (IL)‐4, IL‐5 and IL‐13, drive major features of asthma, including increased eosinophils in the airway or bronchoalveolar lavage fluid, IgE class switching and airway hyper‐responsiveness.^6^ Furthermore, airway epithelial‐derived cytokines IL‐33, IL‐25 and TSLP can potentiate Th2 inflammation.^7^, ^8^ Of interest, patients with Th2 or non‐Th2 inflammation responded differently to anti‐inflammatory treatment, including inhaled corticosteroids.^9^ Patients with non‐Th2 asthma may be less responsive to steroids compared with a predominantly Th2‐high disease. Thus, it is essential to identify markers that could indicate whether patients are Th2 or non‐Th2‐type inflammation, or to identify therapeutic targets that could treat patients with Th2 or non‐Th2‐type inflammation.

Current therapies for asthma include β2‐adrenoreceptor agonists for episodes of acute bronchospasm and corticosteroids for controlling the chronic changes in the disease process. However, β2‐adrenoreceptor agonists do not reverse the disease process and mostly provide symptomatic relief. Moreover, β2‐adrenoreceptor agonists are subject to tachyphylaxis and patients generally develop tolerance requiring higher doses to control symptoms over time.^10^, ^11^ Inhaled corticosteroids are the mainstay therapy for allergic asthma.^12^ Because of their anti‐inflammatory action, corticosteroids can reverse airway inflammation and have a positive effect on the disease pathogenesis. Unfortunately, corticosteroids are unable to revert all changes associated with asthma.^13^ Given the shortcomings of β2‐agonists and inhaled corticosteroids, a variety of novel and potential drug targets have been recently identified, including leukotriene inhibitors, monoclonal antibodies (e.g. omalizumab, mepolizumab, benralizumab and dupilumab),^8^ prostaglandin D2 receptor 2 antagonists (e.g. DP2 antagonist GB001) and RhoA/Rho‐kinase.^14^, ^15^ Of these, omalizumab is the first biological acknowledged by Global Initiative for Asthma (GINA) as add‐on therapy against IgE‐mediated allergic asthma.^16^ Mepolizumab is a humanised monoclonal antibody that targets IL‐5 for the treatment of severe asthma with an eosinophilic phenotype.^17^ Benralizumab is a monoclonal antibody that binds to the α subunit of IL‐5 receptor (IL‐5Rα) against severe eosinophilic asthma.^18^ Dupilumab is a monoclonal antibody directed against the α subunit of the IL‐4 receptor (IL‐4Rα) acting as a dual antagonist of both IL‐4 and IL‐13 for the treatment of severe type 2 asthma.^19^ In addition, the ras homolog family member A (RhoA) of the Rho family GTPases is a nucleotide‐dependent protein, switching between an inactive form, GDP‐bound, and an active form, GTP‐bound RhoA. Upregulated expression and activation of RhoA have been reported in asthma,^20^, ^21^ and studies from our laboratory have demonstrated that inhibition of RhoA/ROCK signalling not only prevented allergic airway inflammation but also reversed established cockroach allergen‐induced airway inflammation and remodelling.^22^ Furthermore, RhoA has also been shown to regulate different biological functions such as cell recruitment, proliferation, differentiation and apoptosis.^22^, ^23^, ^24^ Collectively, these findings suggest that RhoA is a promising and novel therapeutic target for asthma.

Two major Rho‐kinase inhibitors and their analogs have been developed, including fasudil and Y‐27632. Fasudil is also known as HA‐1077 (1‐(5‐isoquinolinesulfonyl)‐homo‐piperazine) and has a high affinity for Rho‐kinase with its inhibition constant of 0.33 μm. It also inhibits myosin light chain kinase, PKC and cAMP‐dependent protein kinase. Fasudil is currently the only Rho‐kinase inhibitor that is commercially available and used for the prevention of vasospasm in patients with subarachnoid haemorrhage in Japan.^25^ Hydroxyfasudil, a metabolite of fasudil, also causes smooth muscle relaxation via the same mechanism. HA‐1152P is another derivative of fasudil which has higher inhibitory potency for Rho‐kinase (K_i_‐ 1.6 nm) and better selectivity profile for PKC, PKA and MLCK, but its related studies are very limited.^26^ Y‐27632 [(+)‐(R)‐trans‐4‐(1‐aminoethyl)‐N‐4‐pyridyl) cyclohexanecarboxamide dihydrochloride] is another commonly used Rho‐kinase inhibitor. It is cell permeable and an ATP‐competitive inhibitor of Rho‐kinase.^27^ Analogs of Y‐27632 include Y‐30141 and Y‐30694. Both have similar activity as Y‐27632, which can inhibit cAMP‐dependent protein kinase at relatively low concentrations.^27^, ^28^ However, none of these were applied for the treatment of asthma.

In this review, we evaluate the literature regarding the recent advances in the regulation of RhoA/Rho‐kinase activation and the role of RhoA/Rho‐kinase in regulating major features of asthma, such as airway hyper‐responsiveness, remodelling and inflammation. We also discuss the newly identified roles of RhoA/Rho‐kinase signalling in MSC differentiation and bronchial epithelial barrier dysfunction.

## Activation of RhoA/Rho‐kinase

**Figure 1:**
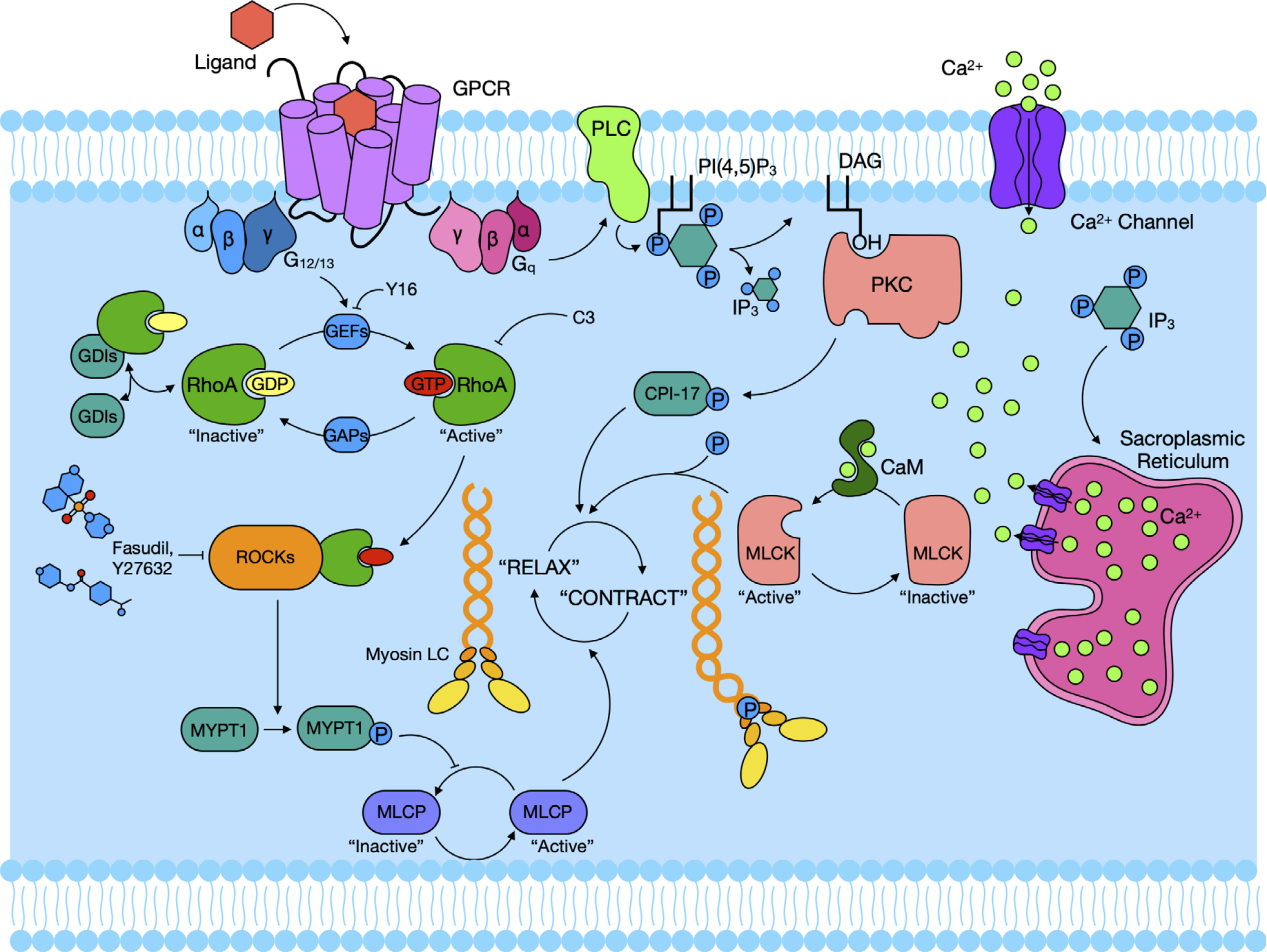
RhoA/Rho‐kinase signalling activation. Upon stimulation by a G protein‐coupled receptor (GPCR) agonist, inactive GDP‐bound RhoA (GDP‐RhoA) is converted into its active state, GTP‐bound RhoA (GTP‐RhoA), by guanine nucleotide exchange factors (GEFs). In turn, activated GTP‐RhoA can also be converted into inactivated GDP‐RhoA y GTPase‐activating proteins (GAPs). Rho‐kinases can lead to the phosphorylation of myosin phosphatase targeting subunit 1 (MYPT1) and exert an inhibitory effect on myosin light chain phosphatase (MLCP) that induces smooth muscle relaxation by the removal of the phosphate on myosin light chain (MLC). The GTP‐bound α subunit of the αβγ holomer dissociates and activates phospholipase C (PLC) increasing intracellular calcium levels via the activity of inositol 1,4,5‐triphosphate (IP3) and diacylglycerol (DAG). IP3 interacts with specific receptors on the sarcoplasmic reticulum leading to an increased cellular Ca^2+^ concentration and subsequently activation of myosin light chain kinase (MLCK) through calmodulin (CaM). CPI‐17 is an inhibitor protein for MLCP and can be activated by protein kinase C (PKC) and promote MLC phosphorylation.

Rho is a monomeric G protein that belongs to the Ras superfamily of proteins with six isoforms (A‐E and G). Of these, isoform A, namely RhoA, is the most studied and has GDP and GTP binding activity as well as GTPase activity.^29^ As shown in Figure 1, upon stimulation by G protein‐coupled receptor (GPCR) agonists, inactive GDP‐RhoA can be converted to its active state, GTP‐RhoA. It is well‐recognised that RhoA activity is mainly modulated by guanine nucleotide exchange factors (GEFs), GTPase‐activating proteins (GAPs) and guanine nucleotide dissociation inhibitors (GDIs).^30^ For example, GEFs stimulate GDP‐GTP exchange process and lead to GTP‐RhoA activation. GAPs cause stimulation of slow intrinsic GTPases and lead to the formation of inactive GDP‐RhoA. GDIs form cytoplasmic complexes with GDP‐RhoA and prevent membrane anchoring.

GTP‐RhoA acts by binding to Rho‐kinase and causes its activation. Rho‐kinase is found in the cytoplasm and moves to plasma membrane in the presence of GTP‐RhoA by binding the Rho‐binding domain (RBD). Upon activation, Rho‐kinase interacts with myosin light chain phosphatase (MLCP) and inhibits its activity by phosphorylation of threonine 696 and 853 of myosin‐binding subunit, myosin phosphatase targeting subunit 1 (MYPT1).^31^ MYPT1 phosphorylation exerts an inhibitory effect on dephosphorylation of MLC limiting the airway smooth muscle relaxation.^14^ Airway smooth muscle contraction induced by muscarinic receptor agonists such as acetylcholine (Ach), methacholine (MCh), histamine, prostaglandins or leukotrienes is one of the major clinical features of asthma. These agonists cause contraction along with increasing intracellular concentrations of Ca^2+^ (Ca^2+^‐dependent mechanisms), initially via Ca^2+^ mobilisation from sarcoplasmic reticulum (SR), and subsequently increasing cellular Ca^2+^ concentration via voltage Ca^2+^ channel.^32^ Additionally, RhoA was activated through cell membrane‐bound G protein‐coupled receptors like G_q_ and G_12/13_ via exchange of α subunit GDP for GTP. Binding of GTP to the α subunit induces dissociation of the αβγ holomer from the α subunit. The GTP‐bound α subunit then activates phospholipase C (PLC) to generate inositol 1,4,5‐triphosphate (IP3) and diacylglycerol (DAG). IP3 interacts with specific receptors on the SR leading to the release of calcium. Calcium forms a Ca^2+^‐calmodulin–myosin light chain kinase (MLCK) complex which activates MLCK, and the activated MLCK phosphorylates myosin light chain (MLC), thus leading to smooth muscle contraction. All these processes are together referred to as a Ca^2+^‐dependent contraction.^33^, ^34^ In conjunction with the Ca^2+^‐dependent contraction, MLC phosphorylation is also regulated by the enzyme MLC phosphatase. Active MLC phosphatase can cause MLC dephosphorylation, leading to smooth muscle relaxation. Additionally, CPI‐17, activated by protein kinase C (PKC), can directly promote MLC phosphorylation by regulating MLCP activation.^35^ MLC phosphorylase is regulated by various intracellular signals via the myosin‐binding subunit. Of these, RhoA/Rho‐kinase is one of the most important and well‐studied intracellular molecules that regulates phosphorylation of the myosin‐binding subunit of MLC phosphatase, thus promoting smooth muscle contraction. Ca^2+^ sensitisation is sensitive to a RhoA inactivator (C3 exoenzyme) and a Rho‐kinase inhibitor (Y‐27632), indicating that RhoA/Rho‐kinase pathway may play a critical role in Ca^2+^ sensitisation. Indeed, RhoA/Rho‐kinase‐mediated Ca^2+^ sensitisation is markedly enhanced in experimental asthma and involved in bronchial smooth muscle (BSM) hyper‐responsiveness.^36^

**Figure 2:**
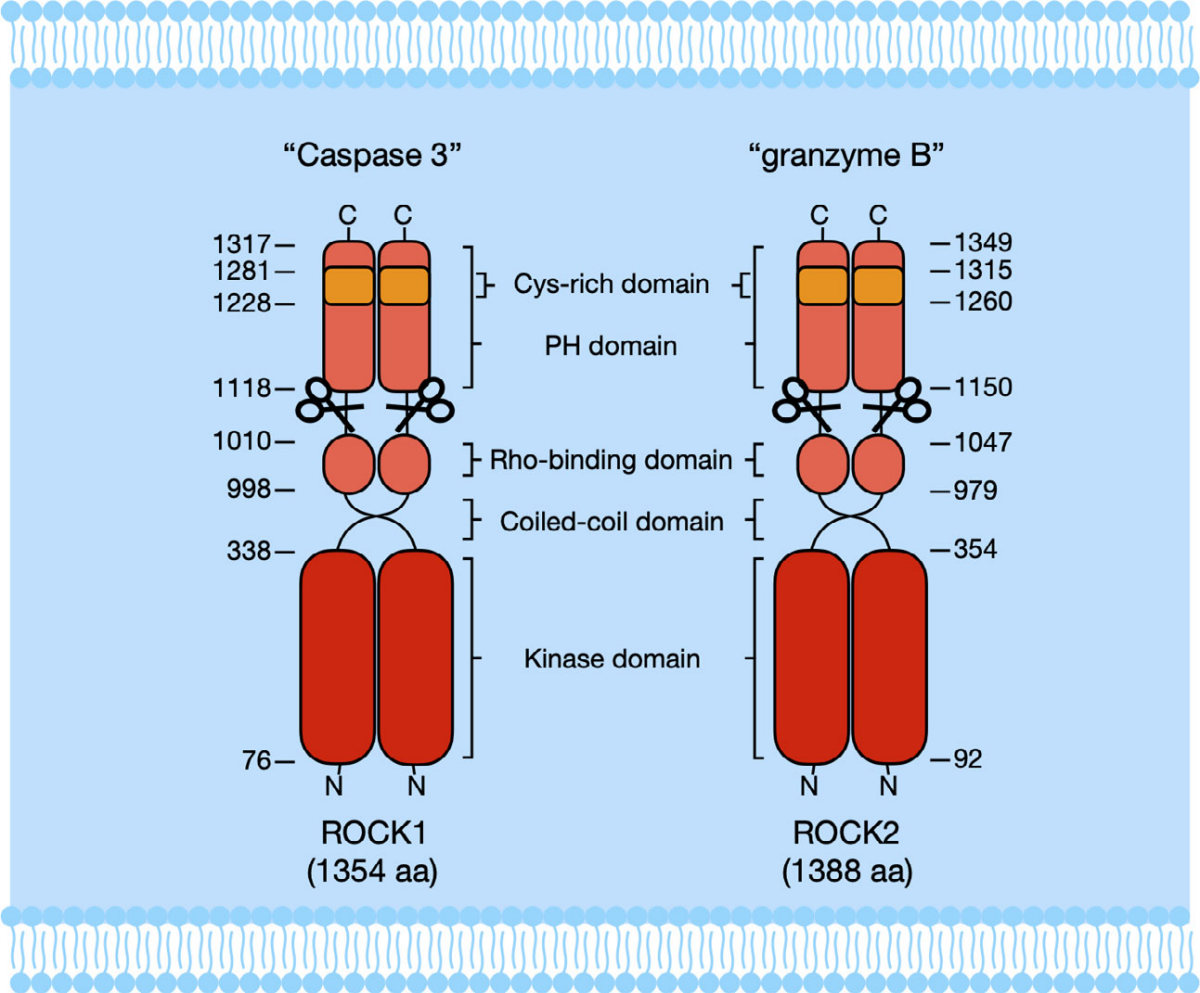
Molecular structure of Rho‐kinase isoforms. Two isoforms of Rho‐kinase (ROCK1 and ROCK2) consist of three major domains, including a kinase domain on the N‐terminal domain, a coiled‐coil domain with Rho‐binding domain in the centre, and a putative pleckstrin homology (PH) domain with the internal cysteine‐rich domain on the C‐terminal domain. Rho‐kinase activation includes that ROCK1 is specifically cleaved by caspase‐3, whereas ROCK2 is cleaved by granzyme B.

Rho‐kinase is also known as ROCK (Rho‐associated coiled‐coil forming kinases) that is a serine/threonine kinase from the AGC (cAMP‐dependent protein kinase A/protein kinase G/protein kinase C) kinase family. It is a 160 kDa protein that has the kinase domain on the N‐terminus, the coiled‐coil domain at the centre (containing Rho‐binding domain) and a pleckstrin homology (PH) domain (containing internal cysteine‐rich domain) on the C‐terminus (Figure 2).^37^ There are two identified Rho‐kinase isoforms: ROCK1 and ROCK2; their kinase domains are highly homologous and conserved, with 65% amino acid sequences in common.^38^ Because of the kinase homology, both isoforms are ubiquitously expressed in human as well as rodent tissues. Therefore, for simplicity both the isoforms are referred to as Rho‐kinases. Rho‐kinase is activated in two different ways through disruption of its auto‐inhibitory intramolecular fold: Rho‐dependent and Rho‐independent activation. Rho‐dependent activation is mediated by combining active GTP‐RhoA with RBD. However, the Rho‐independent activation is mediated by ROCK1 that is cleaved by caspase‐3, but ROCK2 is specifically cleaved by granzyme B.^39^, ^40^

## Cytokine regulation of RhoA/Rho‐kinase activation

**Figure 3:**
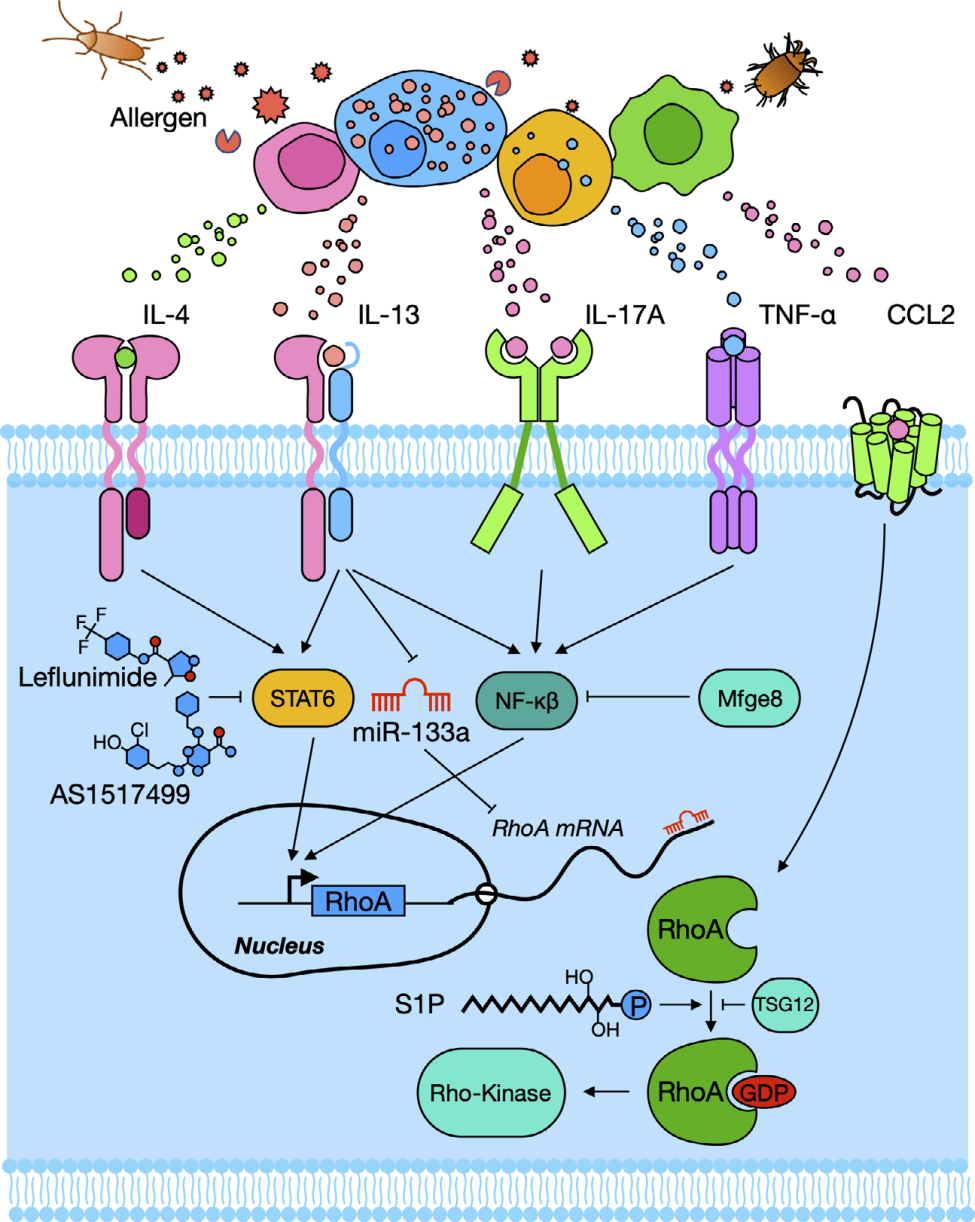
Cytokine and epigenetic regulation of RhoA/Rho‐kinase activation. IL‐4, IL‐13, TNF‐α, IL‐17A and CCL2 released from allergen‐activated immune cells regulate RhoA/Rho‐kinase activation through the bindings of their transcriptional factor STAT6 or NF‐kβ to the promoter regions of RhoA gene. MiR‐133a negatively regulates RhoA expression.

Various transcriptional and post‐transcriptional processes have been shown to regulate the RhoA/Rho‐kinase signalling pathway. Various cytokines have been reported to regulate RhoA expression and activity. As shown in Figure 3, IL‐13, one of the most important Th2 cytokines upregulated RhoA expression in human bronchial smooth muscle cells (hBSMCs) via the activation of the signal transducer and activator of transcription 6 (STAT6) and nuclear factor kappa beta (NF‐κβ).^41^, ^42^ Interestingly, both STAT and NF‐κβ binding elements were found on the promoter region of RhoA gene.^41^ IL‐13 has been shown to upregulate RhoA expression in hBSMCs that were attenuated by leflunomide and AS1517499, inhibitors of STAT6.^42^, ^43^ Similarly, TNF‐α also increased RhoA expression and via NF‐κβ binding elements, leading to bronchial hyper‐responsiveness in asthma.^41^ IL‐4 can induce RhoA mRNA expression and enhance promoter activity of RhoA gene in cultured hBSMCs via STAT6 pathway as well.^44^ IL‐17A can also augment contraction of the airway smooth muscle through activating NF‐κβ‐RhoA‐ROCK2 signalling cascade.^45^ In addition, our recent studies suggest that CCL2 can activate RhoA signalling that contributes to macrophage polarisation and subsequently the development of allergic airway inflammation.^46^

## Epigenetic regulation of RhoA/Rho‐kinase activation

MicroRNAs (miRNAs), specifically miR‐133a, have been recently reported to regulate RhoA protein expression (Figure 3).^47^ miRNAs are small, non‐coding sequences of single‐stranded RNAs that negatively modulate the expression of various genes.^24^, ^46^ They bind to the 3′‐untranslated region (UTR) of the target mRNAs resulting in cleavage of mRNA and repression of translation.^24^ IL‐13 could upregulate RhoA expression by downregulating miR‐133a expression in human BSM.^47^ Recent studies suggest that miR‐142‐5p, as a growth promotive miRNA, plays a significant role in regulating neurogenic differentiation of adipose‐derived stem cells by targeting RhoA/ROCK1.^48^ In addition, miR‐155 plays an important role in TGF‐β‐induced epithelial–mesenchymal transition, cell migration and invasion by downregulating RhoA.^49^ Collectively, these findings reveal that miRNAs may be therapeutic candidates for asthma by targeting RhoA/Rho‐kinase signalling.

## RhoA/Rho‐kinase regulates major features of asthma

Increased activation of RhoA/Rho‐kinase observed in asthmatic patients was associated with bronchial smooth muscle contraction and airway hyper‐responsiveness.^50^ Recent advances have suggested that RhoA is associated not only with airway hyper‐responsiveness,^36^, ^50^ but also with other key features of asthma, such as airway remodelling, allergic airway inflammation and airway barrier dysfunction. Particularly, others and we have recently suggested significant roles for RhoA in regulating allergic airway inflammation and remodelling.^22^, ^51^, ^52^ Here, we reviewed recent advances on the role of RhoA/Rho‐kinase in airway hyper‐responsiveness, remodelling, inflammation and epithelial barrier function.

## RhoA/Rho‐kinase and Airway Hyper‐responsiveness

Increased contractility of airway smooth muscle has been recognised as one of the major risk factors contributing to airway hyper‐responsiveness (AHR). The contraction of airway smooth muscles is through either Ca^2+^‐dependent or Ca^2+^‐independent pathway. RhoA/Rho‐kinase signalling is mainly involved in Ca^2+^‐independent pathway (Ca^2+^ sensitisation). Mechanistically, RhoA/Rho‐kinase regulates MLC phosphatase activity that is critical in removing phosphate from the phosphorylated MLC to induce airway smooth muscle relaxation.^4^ RhoA can also induce actin polymerisation by increasing the globular (F/G) actin ratio^53^. Activated Rho‐kinase indirectly modulates inactivation of actin depolymerisation factor called cofilin, possibly by activation of LIM‐kinase.^54^ Studies with Rho‐kinase inhibitor, Y‐27632, showed that Y‐27632 can relax the isolated human bronchial preparation and inhibit the increased airway resistance.^55^, ^56^, ^57^ Very impressively, the bronchodilatory effect of inhaled Y‐27632 starts promptly and lasts over 8 h.^58^, ^59^ Recently, Fong *et al.* ^59^ firstly reported that ARHGEF12 was the most highly expressed RhoGEF in the airway smooth muscle of asthmatic patients and in the lung tissues of house dust mite (HDM)‐induced mouse model of asthma. The ARHGEF12‐deficient mice showed significantly decreased AHR relative to wild‐type mice.^59^ ARHGEF1, another RhoGEF, is an activator of RhoA increased in the airway smooth muscles from patients with asthma and in the lung tissues from OVA‐challenged mice.^61^ A small interfering RNA (siRNA) against ARHGEF1 attenuated the TGF‐β1‐enhanced RhoA expression, Rho‐kinase activation and airway smooth muscle contraction, respectively.^60^ In addition, TGF‐β1 enhanced basal and methacholine‐induced cytoskeletal stiffness in isolated human airway smooth muscle in a time‐ and dose‐dependent manner.^61^ Importantly, TGF‐β1 was able to induce single‐cell contraction with concomitant increase in phosphorylation of MLC and MYPT1 that was attenuated by pharmacological inhibition of Rho‐kinase^62^. In contrast, TGF‐β1 expression could be regulated by RhoA/Rho‐kinase and PI3K pathway in human airway smooth muscles after stretch treatment.^63^ Together, these studies suggested a positive loop between TGF‐β1 and RhoA/Rho‐kinase signalling that drives the airway hyper‐responsiveness in asthma.

Prostaglandin D_2_ (PGD_2_), one of the major lipid mediators of airway inflammation, could augment contraction of isolated BSM from naïve mice when the tissues were pre‐contracted partially with K^+^. One of the PGD2 receptor antagonists GB001 has been shown to have a rapid and sustained effect on lung function.^15^ However, the PGD_2_‐induced enhancement of contraction was suppressed by using Rho‐kinase inhibitor Y‐27632. This finding suggests that the activation of Rho‐kinase signalling mediated the PGD_2_‐induced BSM hyper‐responsiveness.^64^ Compared to β_2_‐agonists, metallothionein‐2 protein has a better effect in alleviating tension in tracheal spirals and relaxing airway smooth muscles in rodent asthma model.^63^ Further mechanistic studies suggest that TSG12, a TG2 agonist, relaxed airway smooth muscles and reduced asthmatic pulmonary resistance through RhoA phosphorylation in both OVA‐ and HDM‐induced mouse models of asthma.^63^ This study suggests that RhoA plays a critical role in airway smooth muscle relaxation through an important functional axis of RhoA/Rho‐kinase‐MYPT1‐MLC signalling.

Milk fat globule–EGF factor 8 (Mfge8) functions as a cell adhesion protein to connect smooth muscle to elastic fibre in arteries.^64^ Airway biopsies from asthmatic patients showed reduced expression of Mfge8 compared with healthy controls. Mfge8‐deficient mice (Mfge8^−/−^) developed exaggerated AHR in an experimental asthma model,^65^ suggesting that Mfge8 may protect against the exaggerated AHR. Indeed, airway smooth muscles from Mfge8^−/−^ mice showed augmented contraction after treatment with IL‐13, IL‐17A or TNF‐α. In contrast, treatment with recombinant Mfge8 reduced IL‐13‐induced contraction of airway smooth muscles. Further studies suggest one of the important mechanisms that Mfge8 could suppress contraction of airway smooth muscles through inhibiting RhoA.^66^

Sphingosine‐1‐phosphate (S1P: a bioactive lysophospholipid) has been shown to increase the contraction tension in isolated BSM tissues when they are pre‐depolarised by K^+^, and was attenuated by Y‐27632.^67^ Similarly, S1P markedly enhanced contractile force of tracheal smooth muscle in guinea pig, which was also inhibited by Y‐27632. Furthermore, an *in vitro* analysis demonstrated that S1P increased the activation of RhoA and phosphorylation of MYPT1 in cultured human bronchial smooth muscle cells, and pretreatment with Y‐27632 inhibited augmented phosphorylation of MYPT1.^68^ Taken together, these findings indicate that RhoA/Rho‐kinase signalling shows promise for AHR, suggesting that RhoA/Rho‐kinase may be a potential therapeutic target for severe asthma.

## RhoA/Rho‐kinase and airway remodelling

Airway remodelling is characterised by airway smooth muscle proliferation and subepithelial fibrosis. Airway smooth muscle proliferation is mainly due to augmented levels of growth factors that contribute to both cellular hypertrophy and hyperplasia.^69^, ^70^ Subepithelial fibrosis is caused by the deposition of collagen fibrils and proteoglycan beneath the basement membrane along with proliferation of blood vessels leads to airway wall thickening.^70^ Bronchial biopsies from patients with mild to moderate asthma showed smooth muscle cell hyperplasia, and it is well‐documented that airway remodelling is associated with increasing severity of asthma.^71^

Airway smooth muscle cell proliferation is much more rapid upon stimulation in asthmatic patients relative to normal subjects.^72^ Recent advances have suggested that Rho/Rho‐kinase pathway may play a significant role in the process of airway smooth muscle proliferation, and inhibition of the Rho‐kinase pathway has been considered to be a great therapeutic approach for the treatment of airway remodelling in asthma. For example, studies demonstrated an increased expression of RhoA in rats with airway remodelling in asthma.^73^ Y‐27632 along with C3 exoenzyme and simvastatin was also shown to suppress human bronchial smooth muscle cell proliferation because of RhoA activation. Simvastatin, a commonly used HMG‐COA reductase inhibitor, causes attenuation of BSMC via geranylgeranylation of RhoA.^55^ The Rho‐kinase inhibitor Y‐27632 decreased the expression levels of F‐actin and α‐tubulin, suggesting a link for Rho‐kinase and airway smooth muscle cytoskeleton in airway remodelling in asthma.^73^ Furthermore, sphingosylphosphorylcholine (SPC), a sphingolipid metabolite, could stimulate a‐SMA expression in human foetal lung fibroblast and fibroblast‐mediated collagen gel contraction.^74^ Of interest, the increased expression of a‐SMA was blocked by Y‐27632, indicating that RhoA signalling may be involved in SPC‐induced airway remodelling in asthma. We also asked the question whether RhoA signalling is involved in cockroach allergen‐induced airway remodelling in our well‐established chronic mouse model of asthma.^22^ We found increased activation of RhoA signalling in asthmatic lung tissues, and treatment with fasudil, a RhoA/ROCK inhibitor, reversed the airway thickening and collagen deposition/fibrosis caused by repeated allergen exposure. These findings are further supported by several studies that inhibition of RhoA signalling suppresses hyperoxia‐induced pulmonary fibrosis in neonatal rats,^75^ blocks intestinal fibrosis^76^ and attenuates experimental pulmonary fibrosis.^77^ Collectively, these results suggest that RhoA signalling is crucial for allergen‐induced airway remodelling.

## RhoA/Rho‐kinase and airway inflammation

In addition to the importance of RhoA/Rho‐kinase in smooth muscle contraction, airway hyper‐responsiveness and airway remodelling, recent studies suggest that RhoA/Rho‐kinase also plays critical roles in airway inflammation. Especially, RhoA/Rho‐kinase is involved in regulating airway inflammation through affecting migration, differentiation and function of various inflammatory cells (e.g. eosinophils, macrophages, mast cells and T cells) in the pathogenesis of asthma.

### Eosinophils

The mobilisation of eosinophils from bone marrow to peripheral blood and the subsequent infiltration of eosinophils into the airway is one of the major features of allergic asthma.^78^ Eosinophils play a significant role in the pathogenesis of allergic asthma, such as airway hyper‐responsiveness, mucus production and airway remodelling.^79^ Thus, blocking the recruitment of eosinophils into lung tissues is a promising therapeutic intervention for allergic asthma. Indeed, RhoA/Rho‐kinase has been shown to play a critical role in mediating eotaxin induced chemotaxis of eosinophils into the lung tissues.^80^ Pretreatment with RhoA/Rho‐kinase inhibitors resulted in suppression of eosinophil recruitment with reduced levels of IL‐4, IL‐5, IL‐13 and eotaxin in bronchoalveolar lavage.^58^, ^81^ Furthermore, a recent study demonstrated for the first time that protein tyrosine phosphatase 2 (SHP2) and RhoA were robustly activated in the airway eosinophils of children with allergic asthma and of a mouse model with allergic airway inflammation, and SHP2 regulates eosinophil recruitment into lungs through RhoA/ROCK signalling in allergic asthma.^82^ Specifically, inhibition of SHP2 activity reverses the dephosphorylation of p190‐A Rho GTPase‐activating protein and subsequently attenuates activation of RhoA/Rho‐kinase signalling, leading to reduction of eosinophil migration in response to platelet‐activating factor stimulation. Furthermore, genetic interaction between RhoA and SHP2 indicated that RhoA inactivation and SHP2 deletion synergistically attenuated the allergen‐induced eosinophil infiltration into lungs. In contrast, overexpression of active RhoA robustly restored the SHP2 deletion–resultant attenuation of allergen‐induced eosinophil recruitment into lungs. Together, this study suggests that the activation of SHP2 and RhoA/ROCK may play a significant role in regulating eosinophil recruitment in allergic asthma, and blockage of the SHP2‐RhoA signalling pathway represents a promising approach for the treatment of allergic asthma.

### Macrophages

Macrophages are the most abundant immune cells in the lungs and capable of possessing numerous and diverse functions required for homeostasis and defence against pathogens.^83^ Although the origin of lung macrophages remains unclear, lung macrophages include those from either differentiated blood monocytes or the proliferated resident macrophages.^84^ Macrophages are heterogeneous population with a combination of inflammatory (M1 macrophage) and anti‐inflammatory functions (M2 macrophage).^84^ Increased M2 macrophages were noted in allergic asthma.^85^ Thus, molecules regulating macrophage polarisation and function are valuable for the treatment of allergic asthma. We have shown that RhoA signalling is critical in controlling macrophage polarisation through the CCL2/CCR2 axis.^46^ In fact, the activation of RhoA signalling has been previously associated with macrophage polarisation.^86^, ^87^ Our study provided further evidence that CCL2 can activate RhoA signalling that plays a key role in mediating CCL2‐driven macrophage M1 polarisation.^46^ In addition to its role in macrophage polarisation, McPhillips *et al.* ^88^ report that RhoA signalling also plays a significant role in the uptake of apoptotic cells by alveolar macrophages. Furthermore, Moon *et al.* ^89^ also suggest that N‐acetylcysteine induces apoptotic cell clearance by alveolar macrophages through downregulating RhoA/Rho‐kinase pathway, resulting in attenuation of lung inflammation in an *in vivo* mouse model. Thus, these studies suggest a significant role for RhoA/Rho‐kinase pathway in the clearance of apoptotic cells and macrophage polarisation in allergic lung inflammation.

### Mast cells

Mast cells are known to be crucial for the regulation of allergic diseases,^90^ and the IgE receptor (FcεRI)‐mediated mast cell activation is the predominant pathway that is associated with various pathophysiological events in allergic inflammatory diseases.^91^ Studies in our laboratory demonstrated a significant infiltration of mast cells in the lung tissues of asthma mouse model^92^ and in nasal eosinophilic polyps of patients with chronic rhinosinusitis with nasal polyps (CRSwNP).^93^ Interestingly, recent studies provided evidence that RhoA signalling is associated with mast cell activation.^94^ In particular, Sheshachalam *et al.* ^94^ showed that RhoA signalling controls mast cell morphology that transitions to an activated form and granule movement for degranulation. Sibilano *et al.* reported a significant impairment in mast cell degranulation in bone marrow‐derived mast cells (BMMCs) with RhoA knockdown^95^. Of importance, mice with either ROCK1 deficiency or treated with RhoA inhibitor fasudil showed protection against IgE‐mediated allergic response in a passive cutaneous anaphylaxis model.^96^ Although studies are limited, these findings provide a rationale that RhoA/Rho‐kinase is crucial for mast cell activation and mast cell‐associated allergic diseases.

### T cells

It was reported that RhoA plays a significant role in controlling thymus development,^97^ T‐cell activation,^98^ migration and proliferation,^99^ and T‐cell receptor responses.^100^ Recently, Yang *et al.* established a RhoA‐deficient mouse line with RhoA knockout specifically in T cells and made several important findings.^51^, ^52^ Of these, RhoA plays an essential role in coordinating mitochondrial function and thymocyte development.^101^ Furthermore, disruption of RhoA in T cells inhibits T‐cell activation and Th2 differentiation and protects against allergen‐induced airway inflammation through affecting several metabolic pathways, such as glycolysis and oxidative phosphorylation.^52^ Very recently, studies from the same laboratory showed that RhoA is one of the major regulators for Th17 differentiation and effector cytokine secretion through the downregulations of key Th17 transcription factors, Stat3 and Rorγt.^51^ Furthermore, disruption of RhoA in T cells markedly suppressed Th17 response and neutrophil‐involved airway inflammation in an allergen‐induced asthma model. Similarly, Y16, a specific RhoA inhibitor, also suppressed Th17 response and allergic airway inflammation. In addition, Rho‐kinase inhibitor in conjunction with anti‐IL‐17 alleviated airway responsiveness, airway inflammation and remodelling, and oxidative stress in mice with chronic allergic lung inflammation.^102^

## RhoA/Rho‐kinase and mesenchymal stem cells

**Figure 4:**
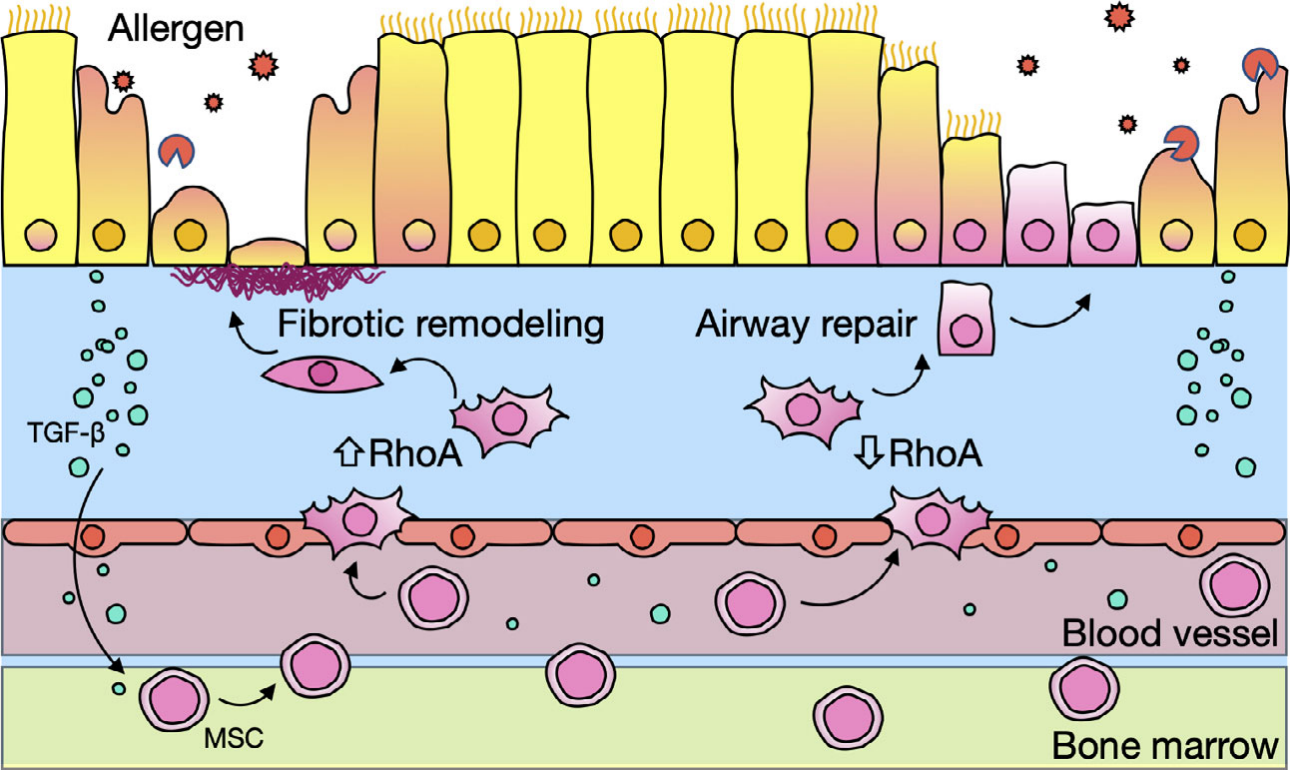
RhoA/Rho‐kinase signalling controls mesenchymal stem cell lineage commitment in asthma. Increased activation of RhoA/Rho‐kinase signalling induces MSC differentiation into fibroblast/myofibroblasts for allergen‐induced airway remodelling. Decreased activation of RhoA/Rho‐kinase signalling induces MSC differentiation towards epithelial cells for airway repairing. TGF‐β1 from damaged airway epithelial cells plays a major role in MSC migration.

Mesenchymal stem cells (MSCs) are progenitor cells that can differentiate into different cell types.^103^ MSCs from bone marrow/circulating blood are able to migrate to the damage sites after tissue injury.^104^, ^105^ We have reported that MSCs were remarkably increased in the lungs of allergen‐sensitised and challenged mice.^106^ TGFβ1‐activated RhoA signalling has been suggested to be a molecular switch for MSC lineage commitment in arterial repair/remodelling after injury.^107^ We also found that RhoA/Rho‐kinase signalling plays a critical role in promoting MSC differentiation (Figure 4).^22^ Specifically, increased activation of RhoA/Rho‐kinase signalling induces MSC differentiation into fibroblast/myofibroblasts for airway remodelling. However, decreased activation of RhoA/Rho‐kinase signalling induces MSC differentiation towards epithelial cells for airway repairing. Mechanistically, we used gene array and identified Lef1 as one of the genes most upregulated in MSCs by RhoA/Rho‐kinase signalling and may be responsible for the RhoA‐mediated MSC differentiation. It was documented that Lef1 is critical in the maintenance of stem cells and organ development, especially in epithelial–mesenchymal transition.^108^ Lef1 is also essential for the transition from mesenchymal to epithelial cells and for Th2 effector differentiation.^109^, ^110^ However, further studies are clearly needed to investigate the underlying mechanisms leading to Lef1 activation by RhoA/Rho‐kinase signalling and downstream components of Lef1 for MSC lineage commitment/differentiation. In addition, the RhoA/Rho‐kinase signalling is also critical in mediating hypoxia‐decreased MSC migration.^111^ Collectively, these findings provide a new understanding of RhoA/Rho‐kinase in controlling MSC migration and lineage commitment.

## RhoA/Rho‐kinase and epithelial barrier function

The disruption and hyper‐permeability of epithelial barrier in the airway and lung are closely associated with the pathogenesis of asthma. RhoA has been considered to be one of the important mechanisms contributing to intestinal and prostate epithelial barrier hyper‐permeability.^112^, ^113^ The mechanisms are also true for cigarette smoke‐induced airway epithelial barrier dysfunction.^114^ Heat shock protein (Hsp) 90α is implicated in the LPS‐induced lung endothelial barrier dysfunction by disrupting RhoA signalling.^115^ Further studies demonstrated increased expression of Hsp90α, E‐cadherin and β‐catenin in cyto‐membrane of HDM‐treated 16HBE cells.^116^ Hsp90α expression was significantly reversed by the inhibition of RhoA, which was paralleled with restored epithelial barrier disruption, suggesting that Hsp90α mediates HDM‐induced epithelial barrier dysfunction partially by the activation of RhoA signalling. In addition, disruption of RhoA/Rho‐kinase also increases actomyosin‐mediated endothelial cell contraction, leading to the loss of endothelial integrity and tissue infiltration of inflammatory cells.^117^ Furthermore, Mikelis *et al.* ^118^ showed that RhoA regulates histamine‐promoted vascular leakage via rapid formation of focal adherent junctions and disruption of endothelial barrier. Interestingly, interfering with RhoA/Rho‐kinase signalling abolished endothelial permeability and passive cutaneous anaphylaxis by using either endothelial RhoA genetic deletion or Rho‐kinase inhibitor fasudil. The findings were further supported by a recent study that Y27632 upregulated p63, gap junction molecules Cx26, Cx30 and Cx43, and downregulated tight junction molecules claudin‐4, claudin‐7 and claudin‐23, leading to epithelial barrier dysfunction.^119^ Together, these findings indicate a key role for the RhoA/Rho‐kinase signalling in bronchial epithelial barrier dysfunction.

## Conclusions

**Figure 5:**
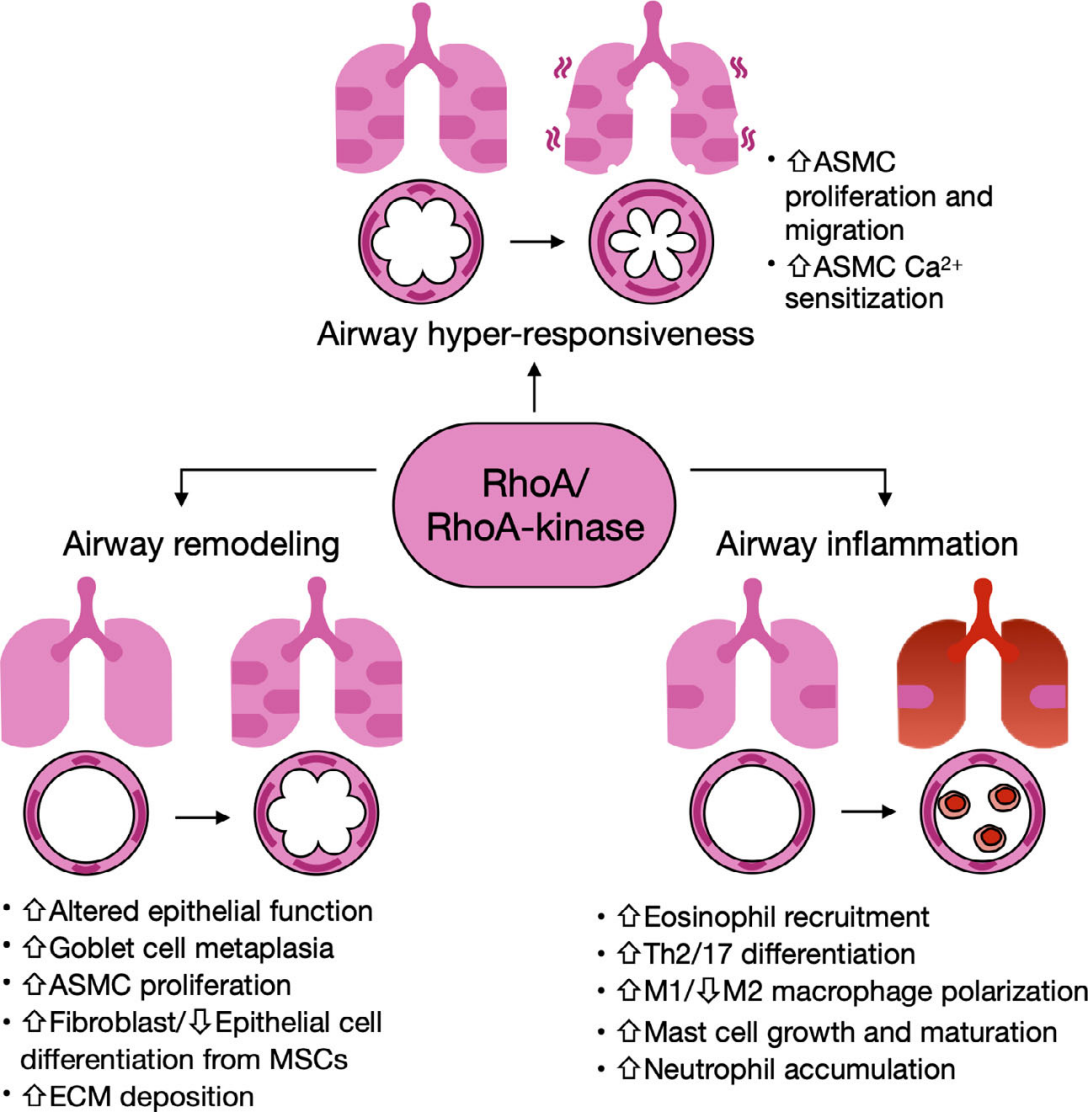
Schematic summary of RhoA/Rho‐kinase regulation on the major characteristics of asthma.

The present review demonstrates that RhoA/Rho‐kinase is substantially involved in the pathophysiological changes of asthma, including airway hyper‐responsiveness, airway remodelling and lung inflammation (Figure 5). Specifically, the activated RhoA/Rho‐kinase pathway affects airway hyper‐responsiveness by promoting cell proliferation and migration, and contractility of airway smooth muscle cell via Ca^2+^ sensitisation. Activated RhoA/Rho‐kinase signalling also contributes to airway remodelling by increasing extracellular matrix deposition and mesenchymal stem cell differentiation into fibroblasts. Furthermore, chronic allergen/environmental pollutant exposure induces airway inflammation through driving inflammatory cell recruitment, differentiation and cytokine release, with all of these major features regulated by RhoA/Rho‐kinase signalling. In addition, RhoA/Rho‐kinase signalling also plays a significant role in regulating MSC differentiation and bronchial epithelial barrier dysfunction. These studies provide evidence that inhibition of RhoA/Rho‐kinase signalling may represent a promising novel therapeutic approach for treatment of asthma. However, most of the data reviewed come from animal models of asthma, and there is still a significant gap between animal models and human diseases. But these studies may provide for important insights into the regulation of major features of asthma and future clinical treatment of diseases. Thus, further studies are essential to provide clinical data, investigate the functional significance of RhoA/Rho‐kinase signalling in asthmatic patients, and determine the underlying mechanisms of RhoA/Rho‐kinase signalling in the regulation of innate immune responses and host defence. Importantly, the development of new, safe, more specific and potent inhibitors targeting RhoA/Rho‐kinase is crucial for the treatment of asthma in the future.

## Conflict of interest

The authors declare no conflict of interest.

## Author Contribution

**Yan Zhang:** Investigation; Writing‐original draft; Writing‐review & editing. **Arjun Saradna:** Investigation; Writing‐original draft; Writing‐review & editing. **Rhea Ratan:** Investigation; Writing‐review & editing. **Xia Ke:** Conceptualization; Investigation; Writing‐review & editing. **Wei Tu:** Investigation; Writing‐original draft. **Danh C Do:** Conceptualization; Investigation; Writing‐original draft; Writing‐review & editing. **Chengping Hu:** Supervision. **Peisong Gao:** Conceptualization; Funding acquisition; Investigation; Supervision; Writing‐original draft; Writing‐review & editing.

## References

[1] Akinbami LJ , Moorman JE , Bailey C *et al* Trends in asthma prevalence, health care use, and mortality in the United States, 2001–2010. NCHS Data Brief 2012; (94): 1–8. 22617340

[2] Sachdeva K , Do DC , Zhang Y , Hu X , Chen J , Gao P . Environmental exposures and asthma development: autophagy, mitophagy, and cellular senescence. Front Immunol 2019; 10: 2787. 31849968 10.3389/fimmu.2019.02787 PMC6896909

[3] Gerthoffer WT , Solway J , Camoretti‐Mercado B . Emerging targets for novel therapy of asthma. Curr Opin Pharmacol 2013; 13: 324–330. 23639507 10.1016/j.coph.2013.04.002 PMC3686997

[4] Schaafsma D , Gosens R , Zaagsma J , Halayko AJ , Meurs H . Rho kinase inhibitors: a novel therapeutical intervention in asthma? Eur J Pharmacol 2008; 585: 398–406. 18410919 10.1016/j.ejphar.2008.01.056

[5] Robinson D , Humbert M , Buhl R *et al* Revisiting Type 2‐high and Type 2‐low airway inflammation in asthma: current knowledge and therapeutic implications. Clin Exp Allergy 2017; 47: 161–175. 28036144 10.1111/cea.12880

[6] Fahy JV . Type 2 inflammation in asthma–present in most, absent in many. Nat Rev Immunol 2015; 15: 57–65. 25534623 10.1038/nri3786 PMC4390063

[7] Boonpiyathad T , Sozener ZC , Satitsuksanoa P , Akdis CA . Immunologic mechanisms in asthma. Semin Immunol 2019; 46: 101333. 31703832 10.1016/j.smim.2019.101333

[8] Agache I , Cojanu C , Laculiceanu A , Rogozea L . Critical points on the use of biologicals in allergic diseases and asthma. Allergy Asthma Immunol Res 2020; 12: 24–41. 31743962 10.4168/aair.2020.12.1.24 PMC6875478

[9] Woodruff PG , Modrek B , Choy DF *et al* T‐helper type 2‐driven inflammation defines major subphenotypes of asthma. Am J Respir Crit Care Med 2009; 180: 388–395. 19483109 10.1164/rccm.200903-0392OC PMC2742757

[10] Wang ZL , Walker BA , Weir TD *et al* Effect of chronic antigen and beta 2 agonist exposure on airway remodeling in guinea pigs. Am J Respir Crit Care Med 1995; 152: 2097–2104. 8520781 10.1164/ajrccm.152.6.8520781

[11] Stewart AG , Harris T , Fernandes DJ *et al* β2‐adrenergic receptor agonists and cAMP arrest human cultured airway smooth muscle cells in the G_1_ phase of the cell cycle: role of proteasome degradation of cyclin D1. Mol Pharmacol 1999; 56: 1079–1086. 10531416 10.1124/mol.56.5.1079

[12] Kearns N , Maijers I , Harper J , Beasley R , Weatherall M . Inhaled corticosteroids in acute asthma: a systemic review and meta‐analysis. J Allergy Clin Immunol Pract 2020; 8: 605–617.e6. 31521830 10.1016/j.jaip.2019.08.051

[13] Vanacker NJ , Palmans E , Kips JC , Pauwels RA . Fluticasone inhibits but does not reverse allergen‐induced structural airway changes. Am J Respir Crit Care Med 2001; 163: 674–679. 11254522 10.1164/ajrccm.163.3.2004160

[14] Chiba Y , Matsusue K , Misawa M . RhoA, a possible target for treatment of airway hyperresponsiveness in bronchial asthma. J Pharmacol Sci 2010; 114: 239–247. 20948164 10.1254/jphs.10r03cr

[15] Ortega H , Fitzgerald M , Raghupathi K *et al* A phase 2 study to evaluate the safety, efficacy and pharmacokinetics of DP2 antagonist GB001 and to explore biomarkers of airway inflammation in mild‐to‐moderate asthma. Clin Exp Allergy 2020; 50: 189–197. 31659803 10.1111/cea.13524 PMC7027764

[16] Djukanovic R , Wilson SJ , Kraft M *et al* Effects of treatment with anti‐immunoglobulin E antibody omalizumab on airway inflammation in allergic asthma. Am J Respir Crit Care Med 2004; 170: 583–593. 15172898 10.1164/rccm.200312-1651OC

[17] Gupta A , Ikeda M , Geng B *et al* Long‐term safety and pharmacodynamics of mepolizumab in children with severe asthma with an eosinophilic phenotype. J Allergy Clin Immunol 2019; 144: 1336–1342.e7. 31425781 10.1016/j.jaci.2019.08.005

[18] Liu T , Wang F , Wang G , Mao H . Efficacy and safety of benralizumab in patients with eosinophilic asthma: a meta‐analysis of randomized placebo‐controlled trials. Front Med 2018; 12: 340–349. 29086236 10.1007/s11684-017-0565-0

[19] Corren J , Castro M , O'Riordan T *et al* Dupilumab efficacy in patients with uncontrolled, moderate‐to‐severe allergic asthma. J Allergy Clin Immunol Pract 2020; 8: 516–526. 31521831 10.1016/j.jaip.2019.08.050

[20] Wang L , Chitano P , Pare PD , Seow CY . Upregulation of smooth muscle Rho‐kinase protein expression in human asthma. Eur Respir J 2020; 55: 1901785. 31727693 10.1183/13993003.01785-2019

[21] Sakai H , Otogoto S , Chiba Y , Abe K , Misawa M . TNF‐alpha augments the expression of RhoA in the rat bronchus. J Smooth Muscle Res 2004; 40: 25–34. 15170075 10.1540/jsmr.40.25

[22] Ke X , Do DC , Li C *et al* Ras homolog family member A/Rho‐associated protein kinase 1 signaling modulates lineage commitment of mesenchymal stem cells in asthmatic patients through lymphoid enhancer‐binding factor 1. J Allergy Clin Immunol 2019; 143: 1560–1574.e6. 30194990 10.1016/j.jaci.2018.08.023 PMC6401351

[23] Loirand G . Rho kinases in health and disease: from basic science to translational research. Pharmacol Rev 2015; 67: 1074–1095. 26419448 10.1124/pr.115.010595

[24] Zhou Y , Do DC , Ishmael FT *et al* Mannose receptor modulates macrophage polarization and allergic inflammation through miR‐511‐3p. J Allergy Clin Immunol 2018; 141: 350–364.e8. 28629744 10.1016/j.jaci.2017.04.049 PMC5944850

[25] Hirooka Y , Shimokawa H . Therapeutic potential of rho‐kinase inhibitors in cardiovascular diseases. Am J Cardiovasc Drugs 2005; 5: 31–39. 15631536 10.2165/00129784-200505010-00005

[26] Sasaki Y , Suzuki M , Hidaka H . The novel and specific Rho‐kinase inhibitor (S)‐(+)‐2‐methyl‐1‐[(4‐methyl‐5‐isoquinoline)sulfonyl]‐homopiperazine as a probing molecule for Rho‐kinase‐involved pathway. Pharmacol Ther 2002; 93: 225–232. 12191614 10.1016/s0163-7258(02)00191-2

[27] Uehata M , Ishizaki T , Satoh H *et al* Calcium sensitization of smooth muscle mediated by a Rho‐associated protein kinase in hypertension. Nature 1997; 389: 990–994. 9353125 10.1038/40187

[28] Ishizaki T , Uehata M , Tamechika I *et al* Pharmacological properties of Y‐27632, a specific inhibitor of rho‐associated kinases. Mol Pharmacol 2000; 57: 976–983. 10779382

[29] Kume H . RhoA/Rho‐kinase as a therapeutic target in asthma. Curr Med Chem 2008; 15: 2876–2885. 18991642 10.2174/092986708786242831

[30] Shimokawa H , Sunamura S , Satoh K . RhoA/Rho‐kinase in the cardiovascular system. Circ Res 2016; 118: 352–366. 26838319 10.1161/CIRCRESAHA.115.306532

[31] Kaibuchi K , Kuroda S , Amano M . Regulation of the cytoskeleton and cell adhesion by the Rho family GTPases in mammalian cells. Annu Rev Biochem 1999; 68: 459–486. 10872457 10.1146/annurev.biochem.68.1.459

[32] Markenscoff‐Papadimitriou E , Allen WE , Colquitt BM *et al* Enhancer interaction networks as a means for singular olfactory receptor expression. Cell 2014; 159: 543–557. 25417106 10.1016/j.cell.2014.09.033 PMC4243057

[33] Snetkov VA , Hapgood KJ , McVicker CG , Lee TH , Ward JP . Mechanisms of leukotriene D4‐induced constriction in human small bronchioles. Br J Pharmacol 2001; 133: 243–252. 11350860 10.1038/sj.bjp.0704076 PMC1572783

[34] Ito S , Kume H , Honjo H *et al* Possible involvement of Rho kinase in Ca^2+^ sensitization and mobilization by MCh in tracheal smooth muscle. Am J Physiol Lung Cell Mol Physiol 2001; 280: L1218–L1224. 11350801 10.1152/ajplung.2001.280.6.L1218

[35] Kitazawa T , Eto M , Woodsome TP , Khalequzzaman M . Phosphorylation of the myosin phosphatase targeting subunit and CPI‐17 during Ca^2+^ sensitization in rabbit smooth muscle. J Physiol 2003; 546: 879–889. 12563012 10.1113/jphysiol.2002.029306 PMC2342583

[36] Chiba Y , Misawa M . The role of RhoA‐mediated Ca^2+^ sensitization of bronchial smooth muscle contraction in airway hyperresponsiveness. J Smooth Muscle Res 2004; 40: 155–167. 15655303 10.1540/jsmr.40.155

[37] Matsui T , Amano M , Yamamoto T *et al* Rho‐associated kinase, a novel serine/threonine kinase, as a putative target for small GTP binding protein Rho. EMBO J 1996; 15: 2208–2216. 8641286 PMC450144

[38] Hahmann C , Schroeter T . Rho‐kinase inhibitors as therapeutics: from pan inhibition to isoform selectivity. Cell Mol Life Sci 2010; 67: 171–177. 19907920 10.1007/s00018-009-0189-x PMC11115778

[39] Coleman ML , Sahai EA , Yeo M , Bosch M , Dewar A , Olson MF . Membrane blebbing during apoptosis results from caspase‐mediated activation of ROCK I. Nat Cell Biol 2001; 3: 339–345. 11283606 10.1038/35070009

[40] Sebbagh M , Hamelin J , Bertoglio J , Solary E , Bréard J . Direct cleavage of ROCK II by granzyme B induces target cell membrane blebbing in a caspase‐independent manner. J Exp Med 2005; 201: 465–471. 15699075 10.1084/jem.20031877 PMC2213043

[41] Goto K , Chiba Y , Matsusue K *et al* The proximal STAT6 and NF‐κB sites are responsible for IL‐13‐ and TNF‐α‐induced RhoA transcriptions in human bronchial smooth muscle cells. Pharmacol Res 2010; 61: 466–472. 20006706 10.1016/j.phrs.2009.12.001 PMC3486725

[42] Chiba Y , Nakazawa S , Todoroki M , Shinozaki K , Sakai H , Misawa M . Interleukin‐13 augments bronchial smooth muscle contractility with an up‐regulation of RhoA protein. Am J Respir Cell Mol Biol 2009; 40: 159–167. 18688040 10.1165/rcmb.2008-0162OC

[43] Chiba Y , Todoroki M , Nishida Y , Tanabe M , Misawa M . A novel STAT6 inhibitor AS1517499 ameliorates antigen‐induced bronchial hypercontractility in mice. Am J Respir Cell Mol Biol 2009; 41: 516–524. 19202006 10.1165/rcmb.2008-0163OC

[44] Chiba Y , Goto K , Momata M , Kobayashi T , Misawa M . Induction of RhoA gene expression by interleukin‐4 in cultured human bronchial smooth muscle cells. J Smooth Muscle Res 2010; 46: 217–224. 20859068 10.1540/jsmr.46.217

[45] Kudo M , Melton AC , Chen C *et al* IL‐17A produced by αβ T cells drives airway hyper‐responsiveness in mice and enhances mouse and human airway smooth muscle contraction. Nat Med 2012; 18: 547–554. 22388091 10.1038/nm.2684 PMC3321096

[46] Do DC , Mu J , Ke X *et al* miR‐511‐3p protects against cockroach allergen‐induced lung inflammation by antagonizing CCL2. JCI Insight 2019; 4: 126832. 31536479 10.1172/jci.insight.126832 PMC6824444

[47] Chiba Y , Tanabe M , Goto K , Sakai H , Misawa M . Down‐regulation of miR‐133a contributes to up‐regulation of Rhoa in bronchial smooth muscle cells. Am J Respir Crit Care Med 2009; 180: 713–719. 19644046 10.1164/rccm.200903-0325OC

[48] Yang L , Wang ZF , Wu H , Wang W . miR‐142‐5p improves neural differentiation and proliferation of adipose‐derived stem cells. Cell Physiol Biochem 2018; 50: 2097–2107. 30415244 10.1159/000495054

[49] Kong W , Yang H , He L *et al* MicroRNA‐155 is regulated by the transforming growth factor β/Smad pathway and contributes to epithelial cell plasticity by targeting RhoA. Mol Cell Biol 2008; 28: 6773–6784. 18794355 10.1128/MCB.00941-08 PMC2573297

[50] Chiba Y , Ueno A , Shinozaki K *et al* Involvement of RhoA‐mediated Ca^2+^ sensitization in antigen‐induced bronchial smooth muscle hyperresponsiveness in mice. Respir Res 2005; 6: 4. 15638941 10.1186/1465-9921-6-4 PMC545934

[51] Yang JQ , Kalim KW , Li Y , Zheng Y , Guo F . Ablation of RhoA impairs Th17 cell differentiation and alleviates house dust mite‐triggered allergic airway inflammation. J Leukoc Biol 2019; 106: 1139–1151. 31260596 10.1002/JLB.3A0119-025RRR PMC7747217

[52] Yang JQ , Kalim KW , Li Y *et al* RhoA orchestrates glycolysis for TH2 cell differentiation and allergic airway inflammation. J Allergy Clin Immunol 2016; 137: 231–245.e4. 26100081 10.1016/j.jaci.2015.05.004 PMC4684821

[53] Togashi H , Emala CW , Hall IP , Hirshman CA . Carbachol‐induced actin reorganization involves Gi activation of Rho in human airway smooth muscle cells. Am J Physiol 1998; 274: L803–L809. 9612296 10.1152/ajplung.1998.274.5.L803

[54] Maekawa M , Ishizaki T , Boku S *et al* Signaling from Rho to the actin cytoskeleton through protein kinases ROCK and LIM‐kinase. Science 1999; 285: 895. 10436159 10.1126/science.285.5429.895

[55] Yoshii A , Iizuka K , Dobashi K *et al* Relaxation of contracted rabbit tracheal and human bronchial smooth muscle by Y‐27632 through inhibition of Ca^2+^ sensitization. Am J Respir Cell Mol Biol 1999; 20: 1190–1200. 10340938 10.1165/ajrcmb.20.6.3441

[56] Hashimoto K , Peebles RS , Sheller JR *et al* Suppression of airway hyperresponsiveness induced by ovalbumin sensitisation and RSV infection with Y‐27632, a Rho kinase inhibitor. Thorax 2002; 57: 524. 12037228 10.1136/thorax.57.6.524 PMC1746359

[57] Iizuka K , Shimizu Y , Tsukagoshi H *et al* Evaluation of Y‐27632, a rho‐kinase inhibitor, as a bronchodilator in guinea pigs. Eur J Pharmacol 2000; 406: 273–279. 11020491 10.1016/s0014-2999(00)00504-5

[58] Henry PJ , Mann TS , Goldie RG . A rho kinase inhibitor, Y‐27632 inhibits pulmonary eosinophilia, bronchoconstriction and airways hyperresponsiveness in allergic mice. Pulm Pharmacol Ther 2005; 18: 67–74. 15607129 10.1016/j.pupt.2004.10.002

[59] Fong V , Hsu A , Wu E *et al* Arhgef12 drives IL17A‐induced airway contractility and airway hyperresponsiveness in mice. JCI Insight 2018; 3: 123578. 30385725 10.1172/jci.insight.123578 PMC6238747

[60] Shaifta Y , MacKay CE , Irechukwu N *et al* Transforming growth factor‐β enhances Rho‐kinase activity and contraction in airway smooth muscle via the nucleotide exchange factor ARHGEF1. J Physiol 2018; 596: 47–66. 29071730 10.1113/JP275033 PMC5746525

[61] Mohamed JS , Boriek AM . Stretch augments TGF‐β1 expression through RhoA/ROCK1/2, PTK, and PI3K in airway smooth muscle cells. Am J Physiol Lung Cell Mol Physiol 2010; 299: L413–L424. 20511342 10.1152/ajplung.90628.2008 PMC2951069

[62] Ojiaku CA , Cao G , Zhu W *et al* TGF‐β1 evokes human airway smooth muscle cell shortening and hyperresponsiveness via Smad3. Am J Respir Cell Mol Biol 2018; 58: 575–584. 28984468 10.1165/rcmb.2017-0247OC PMC5946330

[63] Yin LM , Ulloa L , Yang YQ . Transgelin‐2: biochemical and clinical implications in cancer and asthma. Trends Biochem Sci 2019; 44: 885–896. 31256982 10.1016/j.tibs.2019.05.004 PMC7023894

[64] Larsson A , Peng S , Persson H *et al* Lactadherin binds to elastin–a starting point for medin amyloid formation? Amyloid 2006; 13: 78–85. 16911961 10.1080/13506120600722530

[65] Kudo M , Khalifeh Soltani SM , Sakuma SA *et al* Mfge8 suppresses airway hyperresponsiveness in asthma by regulating smooth muscle contraction. Proc Natl Acad Sci USA 2013; 110: 660–665. 23269839 10.1073/pnas.1216673110 PMC3545777

[66] Chiba Y , Suzuki K , Uechi M *et al* Downregulation of sphingosine‐1‐phosphate receptors in bronchial smooth muscle of mouse experimental asthma. Pharmacol Res 2010; 62: 357–363. 20554039 10.1016/j.phrs.2010.05.005

[67] Kume H , Takeda N , Oguma T *et al* Sphingosine 1‐phosphate causes airway hyper‐reactivity by rho‐mediated myosin phosphatase inactivation. J Pharmacol Exp Ther 2007; 320: 766–773. 17105828 10.1124/jpet.106.110718

[68] Joubert P , Hamid Q . Role of airway smooth muscle in airway remodeling. J Allergy Clin Immunol 2005; 116: 713–716. 16159653 10.1016/j.jaci.2005.05.042

[69] Ramos‐Barbon D , Presley JF , Hamid QA , Fixman ED , Martin JG . Antigen‐specific CD4^+^ T cells drive airway smooth muscle remodeling in experimental asthma. J Clin Invest 2005; 115: 1580–1589. 15902312 10.1172/JCI19711 PMC1088014

[70] Fehrenbach H , Wagner C , Wegmann M . Airway remodeling in asthma: what really matters. Cell Tissue Res 2017; 367: 551–569. 28190087 10.1007/s00441-016-2566-8 PMC5320023

[71] Naureckas ET , Ndukwu IM , Halayko AJ , Maxwell C , Hershenson MB , Solway J . Bronchoalveolar lavage fluid from asthmatic subjects is mitogenic for human airway smooth muscle. Am J Respir Crit Care Med 1999; 160: 2062–2066. 10588629 10.1164/ajrccm.160.6.9903131

[72] Johnson PR , Roth M , Tamm M *et al* Airway smooth muscle cell proliferation is increased in asthma. Am J Respir Crit Care Med 2001; 164: 474–477. 11500353 10.1164/ajrccm.164.3.2010109

[73] Wei B , Shang YX , Li M , Jiang J , Zhang H . Cytoskeleton changes of airway smooth muscle cells in juvenile rats with airway remodeling in asthma and the RhoA/ROCK signaling pathway mechanism. Genet Mol Res 2014; 13: 559–569. 24535884 10.4238/2014.January.22.2

[74] Wang XQ , Mao LJ , Fang QH *et al* Sphingosylphosphorylcholine induces α‐smooth muscle actin expression in human lung fibroblasts and fibroblast‐mediated gel contraction via S1P2 receptor and Rho/Rho‐kinase pathway. Prostaglandins Other Lipid Mediat 2014; 108: 23–30. 24614064 10.1016/j.prostaglandins.2014.02.002

[75] Qi XJ , Ning W , Xu F , Dang HX , Fang F , Li J . Fasudil, an inhibitor of Rho‐associated coiled‐coil kinase, attenuates hyperoxia‐induced pulmonary fibrosis in neonatal rats. Int J Clin Exp Pathol 2015; 8: 12140–12150. 26722398 PMC4680343

[76] Holvoet T , Devriese S , Castermans K *et al* Treatment of intestinal fibrosis in experimental inflammatory bowel disease by the pleiotropic actions of a local rho kinase inhibitor. Gastroenterology 2017; 153: 1054–1067. 28642198 10.1053/j.gastro.2017.06.013

[77] Knipe RS , Probst CK , Lagares D *et al* The rho kinase isoforms ROCK1 and ROCK2 each contribute to the development of experimental pulmonary fibrosis. Am J Respir Cell Mol Biol 2018; 58: 471–481. 29211497 10.1165/rcmb.2017-0075OC PMC5894496

[78] Qiu L , Zhang Y , Do DC *et al* miR‐155 modulates cockroach allergen‐ and oxidative stress‐induced cyclooxygenase‐2 in asthma. J Immunol 2018; 201: 916–929. 29967100 10.4049/jimmunol.1701167 PMC6057819

[79] Landolina NA , Levi‐Schaffer F . Eosinophils as a pharmacological target for the treatment of allergic diseases. Curr Opin Pharmacol 2014; 17: 71–80. 25128782 10.1016/j.coph.2014.07.014

[80] Adachi T , Vita R , Sannohe S *et al* The functional role of rho and rho‐associated coiled‐coil forming protein kinase in eotaxin signaling of eosinophils. J Immunol 2001; 167: 4609–4615. 11591790 10.4049/jimmunol.167.8.4609

[81] Taki F , Kume H , Kobayashi T , Ohta H , Aratake H , Shimokata K . Effects of Rho‐kinase inactivation on eosinophilia and hyper‐reactivity in murine airways by allergen challenges. Clin Exp Allergy 2007; 37: 599–607. 17430358 10.1111/j.1365-2222.2007.02693.x

[82] Xu C , Wu X , Lu M *et al* Protein tyrosine phosphatase 11 acts through RhoA/ROCK to regulate eosinophil accumulation in the allergic airway. FASEB J 2019; 33: 11706–11720. 31361966 10.1096/fj.201900698R PMC6902720

[83] Saradna A , Do DC , Kumar S , Fu QL , Gao P . Macrophage polarization and allergic asthma. Transl Res 2018; 191: 1–14. 29066321 10.1016/j.trsl.2017.09.002 PMC5776696

[84] Epelman S , Lavine KJ , Randolph GJ . Origin and functions of tissue macrophages. Immunity 2014; 41: 21–35. 25035951 10.1016/j.immuni.2014.06.013 PMC4470379

[85] Girodet PO , Nguyen D , Mancini JD *et al* Alternative macrophage activation is increased in asthma. Am J Respir Cell Mol Biol 2016; 55: 467–475. 27248771 10.1165/rcmb.2015-0295OC PMC5070104

[86] Tian L , Li W , Yang L *et al* Cannabinoid receptor 1 participates in liver inflammation by promoting M1 macrophage polarization *via* RhoA/NF‐κB p65 and ERK1/2 pathways, respectively, in mouse liver fibrogenesis. Front Immunol 2017; 8: 1214. 29033935 10.3389/fimmu.2017.01214 PMC5625548

[87] Yang L , Dai F , Tang L , Le Y , Yao W . Macrophage differentiation induced by PMA is mediated by activation of RhoA/ROCK signaling. J Toxicol Sci 2017; 42: 763–771. 29142175 10.2131/jts.42.763

[88] McPhillips K , Janssen WJ , Ghosh M *et al* TNF‐α inhibits macrophage clearance of apoptotic cells via cytosolic phospholipase A2 and oxidant‐dependent mechanisms. J Immunol 2007; 178: 8117–8126. 17548650 10.4049/jimmunol.178.12.8117

[89] Moon C , Lee YJ , Park HJ , Chong YH , Kang JL . N‐acetylcysteine inhibits RhoA and promotes apoptotic cell clearance during intense lung inflammation. Am J Respir Crit Care Med 2010; 181: 374–387. 19965809 10.1164/rccm.200907-1061OC

[90] Amin K . The role of mast cells in allergic inflammation. Respir Med 2012; 106: 9–14. 22112783 10.1016/j.rmed.2011.09.007

[91] Schäfer B , Piliponsky AM , Oka T *et al* Mast cell anaphylatoxin receptor expression can enhance IgE‐dependent skin inflammation in mice. Journal of Allergy and Clinical Immunology 2013; 131: 541–548.e9. 10.1016/j.jaci.2012.05.009 PMC3597773 22728083

[92] Qu J , Do DC , Zhou Y *et al* Oxidized CaMKII promotes asthma through the activation of mast cells. JCI Insight 2017; 2: e90139. 28097237 10.1172/jci.insight.90139 PMC5214090

[93] Wang H , Do DC , Liu J *et al* Functional role of kynurenine and aryl hydrocarbon receptor axis in chronic rhinosinusitis with nasal polyps. J Allergy Clin Immunol 2018; 141: 586–600.e6. 28689792 10.1016/j.jaci.2017.06.013 PMC5937692

[94] Sheshachalam A , Baier A , Eitzen G . The effect of Rho drugs on mast cell activation and degranulation. J Leukoc Biol 2017; 102: 71–81. 28411215 10.1189/jlb.2A0616-279RRR

[95] Sibilano R , Frossi B , Suzuki R , *et al* Modulation of FcεRI‐dependent mast cell response by OX4 0L via Fyn, PI3K, and RhoA. Journal of Allergy and Clinical Immunology. 2012; 130(3): 751–760.e2. 10.1016/j.jaci.2012.03.032 22564682

[96] Kapur R , Shi J , Ghosh J *et al* ROCK1 via LIM kinase regulates growth, maturation and actin based functions in mast cells. Oncotarget 2016; 7: 16936–16947. 26943578 10.18632/oncotarget.7851 PMC4941361

[97] Henning SW , Galandrini R , Hall A , Cantrell DA . The GTPase Rho has a critical regulatory role in thymus development. EMBO J 1997; 16: 2397–2407. 9171353 10.1093/emboj/16.9.2397 PMC1169840

[98] Cantrell DA . GTPases and T cell activation. Immunol Rev 2003; 192: 122–130. 12670400 10.1034/j.1600-065x.2003.00028.x

[99] del Pozo MA , Vicente‐Manzanares M , Tejedor R , Serrador JM , Sanchez‐Madrid F . Rho GTPases control migration and polarization of adhesion molecules and cytoskeletal ERM components in T lymphocytes. Eur J Immunol 1999; 29: 3609–3620. 10556816 10.1002/(SICI)1521-4141(199911)29:11<3609::AID-IMMU3609>3.0.CO;2-S

[100] Corre I , Gomez M , Vielkind S , Cantrell DA . Analysis of thymocyte development reveals that the GTPase RhoA is a positive regulator of T cell receptor responses *in vivo* . J Exp Med 2001; 194: 903–914. 11581313 10.1084/jem.194.7.903 PMC2193481

[101] Zhang S , Konstantinidis DG , Yang JQ *et al* Gene targeting RhoA reveals its essential role in coordinating mitochondrial function and thymocyte development. J Immunol 2014; 193: 5973–5982. 25398325 10.4049/jimmunol.1400839 PMC4258484

[102] Dos Santos TM , Righetti RF , Camargo LDN *et al* Effect of anti‐IL17 antibody treatment alone and in combination with rho‐kinase inhibitor in a murine model of asthma. Front Physiol 2018; 9: 1183. 30233389 10.3389/fphys.2018.01183 PMC6134017

[103] Bianco P , Cao X , Frenette PS *et al* The meaning, the sense and the significance: translating the science of mesenchymal stem cells into medicine. Nat Med 2013; 19: 35–42. 23296015 10.1038/nm.3028 PMC3998103

[104] Barbash IM , Chouraqui P , Baron J *et al* Systemic delivery of bone marrow‐derived mesenchymal stem cells to the infarcted myocardium: feasibility, cell migration, and body distribution. Circulation 2003; 108: 863–868. 12900340 10.1161/01.CIR.0000084828.50310.6A

[105] Ortiz LA , Dutreil M , Fattman C *et al* Interleukin 1 receptor antagonist mediates the antiinflammatory and antifibrotic effect of mesenchymal stem cells during lung injury. Proc Natl Acad Sci USA 2007; 104: 11002–11007. 17569781 10.1073/pnas.0704421104 PMC1891813

[106] Gao P , Zhou Y , Xian L *et al* Functional effects of TGF‐β1 on mesenchymal stem cell mobilization in cockroach allergen‐induced asthma. J Immunol 2014; 192: 4560–4570. 24711618 10.4049/jimmunol.1303461 PMC4039654

[107] Li C , Zhen G , Chai Y *et al* RhoA determines lineage fate of mesenchymal stem cells by modulating CTGF‐VEGF complex in extracellular matrix. Nat Commun 2016; 7: 11455. 27126736 10.1038/ncomms11455 PMC4855537

[108] Santiago L , Daniels G , Wang D , Deng FM , Lee P . Wnt signaling pathway protein LEF1 in cancer, as a biomarker for prognosis and a target for treatment. Am J Cancer Res 2017; 7: 1389–1406. 28670499 PMC5489786

[109] Boras‐Granic K , Chang H , Grosschedl R , Hamel PA . Lef1 is required for the transition of Wnt signaling from mesenchymal to epithelial cells in the mouse embryonic mammary gland. Dev Biol 2006; 295: 219–231. 16678815 10.1016/j.ydbio.2006.03.030

[110] Carr T , Krishnamoorthy V , Yu S , Xue HH , Kee BL , Verykokakis M . The transcription factor lymphoid enhancer factor 1 controls invariant natural killer T cell expansion and Th2‐type effector differentiation. J Exp Med 2015; 212: 793–807. 25897173 10.1084/jem.20141849 PMC4419352

[111] Raheja LF , Genetos DC , Wong A , Yellowley CE . Hypoxic regulation of mesenchymal stem cell migration: the role of RhoA and HIF‐1α. Cell Biol Int 2011; 35: 981–989. 21574962 10.1042/CBI20100733

[112] Tong J , Wang Y , Chang B , Zhang D , Liu P , Wang B . Activation of RhoA in alcohol‐induced intestinal barrier dysfunction. Inflammation 2013; 36: 750–758. 23361851 10.1007/s10753-013-9601-7

[113] Tong J , Wang Y , Chang B , Zhang D , Wang B . Evidence for the involvement of RhoA signaling in the ethanol‐induced increase in intestinal epithelial barrier permeability. Int J Mol Sci 2013; 14: 3946–3960. 23429187 10.3390/ijms14023946 PMC3588079

[114] Forteza RM , Casalino‐Matsuda SM , Falcon NS , Valencia Gattas M , Monzon ME . Hyaluronan and layilin mediate loss of airway epithelial barrier function induced by cigarette smoke by decreasing E‐cadherin. J Biol Chem 2012; 287: 42288–42298. 23048036 10.1074/jbc.M112.387795 PMC3516772

[115] Joshi AD , Dimitropoulou C , Thangjam G *et al* Heat shock protein 90 inhibitors prevent LPS‐induced endothelial barrier dysfunction by disrupting RhoA signaling. Am J Respir Cell Mol Biol 2014; 50: 170–179. 23972231 10.1165/rcmb.2012-0496OC PMC3930930

[116] Dong H‐M , Le Y‐Q , Wang Y‐H *et al* Extracellular heat shock protein 90α mediates HDM‐induced bronchial epithelial barrier dysfunction by activating RhoA/MLC signaling. Respir Res 2017; 18: 111–111. 28558721 10.1186/s12931-017-0593-y PMC5450201

[117] Saito H , Minamiya Y , Saito S , Ogawa J‐I . Endothelial Rho and Rho kinase regulate neutrophil migration via endothelial myosin light chain phosphorylation. J Leukoc Biol 2002; 72: 829–836. 12377953

[118] Mikelis CM , Simaan M , Ando K *et al* RhoA and ROCK mediate histamine‐induced vascular leakage and anaphylactic shock. Nat Commun 2015; 6: 6725–6725. 25857352 10.1038/ncomms7725 PMC4394241

[119] Kaneko Y , Konno T , Kohno T *et al* Induction of airway progenitor cells via p63 and KLF11 by Rho‐kinase inhibitor Y27632 in hTERT‐human nasal epithelial cells. Am J Transl Res 2019; 11: 599–611. 30899365 PMC6413250

